# High-quality antidepressant prescribing: please consider whether “perfection is the enemy of progress”

**DOI:** 10.1186/s12916-020-01621-x

**Published:** 2020-05-21

**Authors:** John T. Walkup, Jeffrey R. Strawn

**Affiliations:** 1 Pritzker Department of Psychiatry and Behavioral Health, Anne and Robert H. Lurie Children’s Hospital of Chicago, Northwestern University Feinberg School of Medicine, 225 E. Chicago Ave, Box 10, Chicago, IL 60611 USA; 2 Department of Psychiatry, College of Medicine, University of Cincinnati, Cincinnati, OH USA; 3 Department of Pediatrics, College of Medicine, University of Cincinnati, Cincinnati, OH USA; 4 Division of Child & Adolescent Psychiatry and Division of Clinical Pharmacology, Cincinnati Children’s Hospital Medical Center, Cincinnati, OH USA

**Keywords:** Antidepressants, Pediatrics, Psychiatry, Prevalence, Anxiety disorders, Depression, NICE guidelines

## Background

We read with interest the report by Jack et al. in this issue of *BMC Medicine* [1] regarding adherence to the NICE antidepressant prescribing guidelines for children and young people with depression [2]. Using an extensive database, the authors determined how often children and adolescents were prescribed antidepressants without a recent assessment by a child and adolescent psychiatrist or pediatrician. The results indicate only 25% of children prescribed selective serotonin reuptake inhibitors (SSRIs) had seen a child and adolescent psychiatrist. The paper’s premise is that adherence to the NICE guidelines is important and that the adherence gap is problematic. The authors discuss a number of factors that contribute to the gap, e.g., long waits for specialist care, and some potential solutions including better training for general practitioners who are likely to prescribe. While we support high-quality prescribing [3], we are concerned with the growing number of studies like Jack et al. [1], which suggest the need for more restrictive antidepressant use. Our concern is that such studies, while focused on quality prescribing, may inhibit access to evidence-based care for the large numbers of children affected with anxiety and depression.

## Problems with the current literature regarding antidepressant prescribing

In the USA, like in the UK, an increasing number of reports appear to support restricting antidepressant use in children and young people. Such papers come in a number of forms and have obvious limitations:

- *Reports, often with alarm, of the increasing rates of diagnosis and antidepressant use* [4]. While notable, increasing rates of psychiatric diagnoses and use of evidence-based treatments are to be expected, given our evolving social capacity to address more complex issues affecting children and the expanding evidence-base.
- *Reports on how often antidepressants are prescribed without an appropriate diagnosis* [1, 4]. While it is possible that the lack of an appropriate diagnosis reflects poor medical practice, we do not have much confidence in our medical record systems to accurately capture treatment decision-making.
- *Reports that emphasize the potential harms of antidepressants, underestimate their potential benefit, and do not acknowledge the lack of any real evidence-based alternatives* [5]. Available antidepressants are not perfect, but are much better than the prior generation. Emphasizing the association between antidepressants and suicidal beahvior, when there is no known mechanism, and ignoring the role of the disorders themselves [6] and important environmental factors such as peer and family relationships is a greater problem and distorts the relative risk of antidepressants on suicidal thinking and behavior (NNH = 100–200) [7].
- *Meta-analyses of admittedly poor-quality trials that set benchmarks for clinical care.* We have argued that meta-analyses with large number of poor-quality trials “wash out” what can be learned from the few available high-quality trials [8].

## More, not less, high-quality prescribing is needed

We agree with Jack et al. that there is a problem with the current use of evidence-based medication treatments, but rigid adherence to treatment guidelines does not solve this problem. Rather, the real problem is limited access to care and low treatment prevalence rates; these data should compel us to reconsider the value of research whose apparent goal is to restrict access to evidence-based medication and stigmatize care provided by available and trusted providers such as general practitioners. For example, the NHS documented rates of emotional disorders of 8.1% (mostly, anxiety 7.2% and depression 2.1%) in 5–19-year-olds [9]. In this population, 25.2% had contact with a mental health professional (psychologist or psychiatrist), 33.4% had contact with primary care provider, and 24.1% had no supportive contacts in the past year. Importantly, for our discussion, only 15% of affected children and young people were treated with medication, a rate somewhat lower than the rate in the USA [10]. The NHS report did not detail the type of medication, only noting that older adolescents with emotional disorders were receiving antidepressants. Given the antidepressant response rate of 50–60% for anxiety and depression, it is important to consider whether medication treatments are actually underutilized in this population.

Given the negative long-term sequelae of anxiety and depression (i.e., risk of suicide, educational underachievement, impaired parent and peer relationships, increased risk of developing secondary mental health disorders such as substance use), should we be satisfied with the current rates of access and treatment? Probably not, so to address this substantial gap in care, policymakers and standard-setting bodies must advocate for population mental health strategies that improve early recognition and treatment in primary care including screening, prevention for those at risk, and preemptive care for the early symptomatic and more (not less) high-quality medication treatment for those children and adolescents with conditions with known pharmacological responsiveness. These efforts could ultimately lower the population mental health burden and reserve specialist care for children and adolescents who have not benefited from initial care in general practice.

## What to do?

In writing this commentary, we are reminded of a quote attributed to Winston Churchill—“Perfection is the enemy of progress.” There is a fundamental tension in child and adolescent psychiatry between setting high standards for care and the population health approach. Key to policymaking and resource allocation will be setting a goal for the percentage of children with anxiety and depression who (1) can be treated by general practitioners, (2) must be treated by a child and adolescent psychiatrist, and importantly, (3) should receive psychotherapy and evidence-based antidepressants. We think that most readers will agree that there is an enormous mental health burden and our current systems are not up to the task. We also suspect many readers will agree with us that 100% of children with a mental health problem should have ready access to a good assessment and evidence-based care. We are a long way from that goal, but refocusing on systems change and creating concrete goals will do much to advance the public mental health agenda. Answering the following questions will begin the dialog and get us oriented in the right direction. What proportion of children and young people with anxiety and depression should be assessed by a mental health professional? Is 25.2% the right number? And what proportion should get evidence-based treatment, including an antidepressant medication? If 15% is too low a rate of antidepressant prescribing, what is the optimal proportion that would allow us to conclude that our mental health systems had met the need and closed the gap?

## Data Availability

Not applicable

## References

[1] Jack RH, Joseph RM, Coupland C (2020). Secondary care specialist visits made by children and young people prescribed antidepressants in primary care: a descriptive study using the QResearch database. BMC Med.

[2] NICE, National Institute for Health and Care Excellence. Depression in children and young people: identification and management (CG28). Nice Publications. 2005. https://www.nice.org.uk/guidance/cg28. Accessed 7 May 2020. 31577402

[3] Walkup J (2009). Practice parameter on the use of psychotropic medication in children and adolescents. J Am Acad Child Adolesc Psychiatry.

[4] Sarginson J, Webb RT, Stocks SJ, Esmail A, Garg S, Ashcroft DM (2017). Temporal trends in antidepressant prescribing to children in UK primary care, 2000–2015. J Affect Disord.

[5] Spielmans GI, Spence-Sing T, Parry P (2020). Duty to warn: antidepressant black box suicidality warning is empirically justified. Front Psychiatry.

[6] Bridge JA, Reynolds B, McBee-Strayer SM, Sheftall AH, Ackerman J, Stevens J (2015). Impulsive aggression, delay discounting, and adolescent suicide attempts: effects of current psychotropic medication use and family history of suicidal behavior. J Child Adolesc Psychopharmacol.

[7] Bridge JA, Iyengar S, Salary CB, Barbe RP, Birmaher B, Pincus HA (2007). Clinical response and risk for reported suicidal ideation and suicide attempts in pediatric antidepressant treatment. JAMA..

[8] Walkup JT. Treatment of depressed adolescents. Am J Psychiatr. 2010. 10.1176/appi.ajp.2010.10040566 20595423

[9] NHS Digital (2018). Mental health of children and young people in England, 2017.

[10] Mojtabai R., Olfson M., Han B. (2016). National Trends in the Prevalence and Treatment of Depression in Adolescents and Young Adults. PEDIATRICS.

